# Resolution of Diplopia and Torsion Following Yttrium Aluminum Garnet (YAG) Laser Capsulotomy in a Patient With the Ocular Tilt Reaction

**DOI:** 10.7759/cureus.16443

**Published:** 2021-07-17

**Authors:** Danny Lam, Oliver Chen, Elizabeth L Wong, Ian C Francis

**Affiliations:** 1 Department of Ophthalmology, Sydney Hospital and Sydney Eye Hospital, Sydney, AUS; 2 Department of Ophthalmology, University of New South Wales, Sydney, AUS; 3 Department of Ophthalmology, Prince of Wales Hospital, Sydney, AUS

**Keywords:** ocular tilt reaction, yag laser capsulotomy, neuro-ophthalmology, diplopia, ocular torsion

## Abstract

The ocular tilt reaction is a rare neuro-ophthalmological phenomenon commonly occurring due to an injury to the vestibulo-ocular pathway, or a thalamic, brainstem, or cerebellar lesion. Most ocular tilt reactions are transient and demonstrate spontaneous recovery. This report documents the immediate resolution of diplopia and the patient’s ocular tilt reaction following visual recovery from left yttrium aluminum garnet laser capsulotomy.

## Introduction

The ocular tilt reaction (OTR) is an acquired vertical misalignment (skew deviation) and conjugate rotation (cyclotorsion) of the eyes with ipsilateral head and neck tilt [1]. The OTR indicates an injury to the vestibulo-ocular pathways, commonly a unilateral peripheral deficit of the otolithic input, and may occur with any lesion of the brainstem or cerebellum or with vestibular neuritis [2,3]. The OTR may also relate to a unilateral lesion or infarction of the graviceptive (gravity-related control of body position) brainstem pathways from the vestibular nucleus to the interstitial nucleus of Cajal in the rostral midbrain [2,4].

The main symptom of the OTR occurs when a patient with a head tilt has the head moved back to the vertical position, resulting in diplopia and a perceived tilt in the opposite direction [1]. Most OTRs are transient with spontaneous recovery. However, prism therapy can be utilized for symptomatic relief of vertical diplopia [1].

The differential diagnosis of OTR includes all the causes of vertical squint. Many of these have specific features which allow easy and rapid clinical diagnosis. For example, a third nerve palsy has characteristic ptosis, dilated pupil, and limited ocular rotations. In this report, the authors report a case of an OTR in an elderly man successfully treated with yttrium aluminum garnet (YAG) laser capsulotomy.

This work was conducted in accordance with the National Statement on Ethical Conduct in Human Research and is consistent with the principles that have their origin in the Declaration of Helsinki. Written informed consent was obtained to reveal protected health information of the patient in this case report.

## Case presentation

A 78-year-old man presented with a four-month history of binocular diplopia. He reported that horizontal and vertical lines appeared “tilted” when looking through the left eye compared with the right eye. His previous ophthalmological history included bilateral pseudophakia eight years prior, and a right YAG laser posterior capsulotomy performed three months prior to presentation. He had photographs from his teenage school years which documented a modest left head tilt. His general medical history included a significant head injury at six years of age, mild aortic regurgitation, and bilateral hearing loss for many years. He had a 20-pack-year history of smoking, having quit 45 years previously.

On examination, his visual acuity was 6/6 in the right eye and 6/9 in the left, with intraocular pressures of 16 mmHg in the right eye and 17 mmHg in the left. Slit-lamp examination was normal, apart from moderate posterior capsular opacification in the left eye.

His pupils were normal and briskly reactive to light bilaterally, with no relative afferent pupil defect. Color vision was intact on Ishihara testing for each eye. Ocular motility was normal, with no evidence of fourth nerve palsy. His confrontation fields were normal, and a detailed cranial nerve examination was normal, apart from his pre-existing bilateral sensorineural hearing loss.

Dilated fundus examination with binocular indirect ophthalmoscopy demonstrated an intermittent, 5-10 degree excyclotorsion of the left eye. Cover test in primary gaze demonstrated a left hypotropia at distance and near. A left head tilt was noted. The Bielschowsky three-step test was negative. On TNO testing, stereopsis was less than 480 seconds of arc. Maddox Rod testing was used to verify the patient’s subjective ocular torsion.

Because of the purported OTR, magnetic resonance imaging (MRI) of the brain and orbits was carried out, demonstrating nonspecific high T2 signal foci in the white matter in keeping with moderate small vessel ischemia. However, no specific lesions were found in the thalamus, brainstem, or cerebellum.

Based on the symptomatology and findings, the patient was diagnosed with a left OTR. There was an isolated skew deviation, an abnormal head posture to the left, and intermittent excyclotorsion of the left eye.

To attempt to re-establish stereopsis and improve fusion, the patient underwent a left YAG laser posterior capsulotomy. Immediately after the procedure, TNO testing demonstrated improved stereopsis to 120 seconds of arc. Subjective refraction resulted in visual acuity of 6/5 now being achieved in the left eye.

On review one week later, the patient’s stereopsis had improved further to 60 seconds of arc on TNO. There was a subtle residual right hyperphoria for distance, but he was orthophoric for near. Repeat fundus examination with binocular indirect ophthalmoscopy demonstrated no ocular torsion. However, there was still a residual mild left head tilt.

## Discussion

The OTR is a rare neuro-ophthalmological phenomenon. Table 1 documents a list of the common causes of vertical diplopia, which are differential diagnoses for the OTR [5]. In this case, the patient’s resolution of ocular torsion symptomatology is likely to have represented an otolithic disorder as his MRI demonstrated no specific thalamic, brainstem, or cerebellar lesions.

**Table 1:** Differential diagnoses of vertical diplopia. Diagnoses are presented in order of decreasing frequency.

Common	Rare
Superior oblique palsy	Extraocular muscle trauma from retrobulbar anesthetic injections for cataract surgery
Third nerve palsy	Brown syndrome
Ocular myasthenia gravis	Progressive external ophthalmoplegia
Thyroid orbitopathy	Orbital myositis
Orbital blowout fracture	Ocular neuromyotonia
Orbital hemorrhage (post-traumatic or spontaneous)	Congenital cranial dysinnervation disorders including orbital fibrosis
Orbital inflammatory disease	Isolated inferior oblique palsy
Aberrant regeneration of the third nerve	
Skew deviation (neurological brainstem dysfunction)	

The patient had no other history of acute vestibulocochlear dysfunction, and his deafness was mild, bilateral, and chronic, rather than acute and unilateral. Goto et al. reported a 45-year-old male who developed OTR following an acute vestibulocochlear syndrome which resolved spontaneously [6]. These authors considered that the specific vestibular lesion may have been neurovascular as there was no MRI evidence of thalamic, brainstem, or cerebellar ischemia.

The patient in this case study may have had a similar vestibulocochlear event to the case described above, with their OTR resulting from a peripheral vestibular dysfunction; however, this was not clinically obvious. Regardless, the patient’s OTR findings returned immediately to normal following visual recovery after left YAG laser capsulotomy, suggesting a link between OTR and binocularity.

The authors consider that the rapid resolution of the patient’s OTR occurred due to the immediate improvement of his binocularity. On the other hand, the authors could not exclude that the resolution of the patient’s OTR may have been coincidental. Nevertheless, the time course of the resolution of the patient’s OTR following the left YAG laser capsulotomy suggested a compelling association.

While the patient’s mild residual left head tilt remained, this could well have persisted given that he had a left head tilt, documented photographically, from his teenage years at school. It is possible that this represented a largely resolved fourth nerve palsy dating from his head injury at the age of six, but there were no other features of this other than his mild left head tilt. Abnormal head postures can also occur because of habit rather than intrinsic ophthalmological, neurological, or musculoskeletal disorders [7].

## Conclusions

In conclusion, this elderly patient with diplopia and OTR was now orthophoric, with marked improvements in visual acuity and stereopsis following left YAG laser capsulotomy. His subjective ocular torsion had resolved, and there was no visible residual ocular torsion on binocular indirect ophthalmoscopy. The rapid resolution of the OTR was likely due to the improvement in binocularity, suggesting a further avenue of clinical examination and management options in the treatment of OTR.
